# Exacerbation of Nanoparticle-Induced Acute Pulmonary Inflammation in a Mouse Model of Metabolic Syndrome

**DOI:** 10.3389/fimmu.2020.00818

**Published:** 2020-05-07

**Authors:** Saeed Alqahtani, Lisa M. Kobos, Li Xia, Christina Ferreira, Jackeline Franco, Xuqin Du, Jonathan H. Shannahan

**Affiliations:** ^1^School of Health Sciences, College of Health and Human Sciences, Purdue University, West Lafayette, IN, United States; ^2^National Center for Pharmaceuticals, Life Science and Environment Research Institute, King Abdulaziz City for Science and Technology (KACST), Riyadh, Saudi Arabia; ^3^Purdue Metabolite Profiling Facility, Purdue University, West Lafayette, IN, United States; ^4^Department of Comparative Pathobiology, College of Veterinary Medicine, Purdue University, West Lafayette, IN, United States; ^5^Department of Occupational Medicine and Toxicology, Beijing ChaoYang Hospital, Capital Medical University, Beijing, China

**Keywords:** nanotoxicology, silver nanoparticles, acute inflammation, lipid mediators of inflammatory resolution, statin, susceptibility

## Abstract

Nanotechnology has the capacity to revolutionize numerous fields and processes, however, exposure-induced health effects are of concern. The majority of nanoparticle (NP) safety evaluations have been performed utilizing healthy models and have demonstrated the potential for pulmonary toxicity. A growing proportion of individuals suffer diseases that may enhance their susceptibility to exposures. Specifically, metabolic syndrome (MetS) is increasingly prevalent and is a risk factor for the development of chronic diseases including type-2 diabetes, cardiovascular disease, and cancer. MetS is a combination of conditions which includes dyslipidemia, obesity, hypertension, and insulin resistance. Due to the role of lipids in inflammatory signaling, we hypothesize that MetS-associated dyslipidemia may modulate NP-induced immune responses. To examine this hypothesis, mice were fed either a control diet or a high-fat western diet (HFWD) for 14-weeks. A subset of mice were treated with atorvastatin for the final 7-weeks to modulate lipids. Mice were exposed to silver NPs (AgNPs) via oropharyngeal aspiration and acute toxicity endpoints were evaluated 24-h post-exposure. Mice on the HFWD demonstrated MetS-associated alterations such as increased body weight and cholesterol compared to control-diet mice. Cytometry analysis of bronchoalveolar lavage fluid (BALF) demonstrated exacerbation of AgNP-induced neutrophilic influx in MetS mice compared to healthy. Additionally, enhanced proinflammatory mRNA expression and protein levels of monocyte chemoattractant protein-1, macrophage inflammatory protein-2, and interleukin-6 were observed in MetS mice compared to healthy following exposure. AgNP exposure reduced mRNA expression of enzymes involved in lipid metabolism, such as arachidonate 5-lipoxygenase and arachidonate 15-lipoxygenase in both mouse models. Exposure to AgNPs decreased inducible nitric oxide synthase gene expression in MetS mice. An exploratory lipidomic profiling approach was utilized to screen lipid mediators involved in pulmonary inflammation. This assessment indicates the potential for reduced levels of lipids mediators of inflammatory resolution (LMIR) in the MetS model compared to healthy mice following AgNP exposure. Statin treatment inhibited enhanced inflammatory responses as well as alterations in LMIR observed in the MetS model due to AgNP exposure. Taken together our data suggests that MetS exacerbates the acute toxicity induced by AgNPs exposure possibly via a disruption of LMIR leading to enhanced pulmonary inflammation.

## Introduction

Metabolic syndrome (MetS) is an increasingly prevalent condition representing an emerging issue for both United States and global public health. It is clinically diagnosed by an individual exhibiting three or more of the following components (1) central obesity (increased waist circumference), (2) hypertension, (3) hyperglycemia/insulin resistance, (4) high triglycerides, and (5) reduced high-density lipoprotein (HDL). MetS is a risk factor and precursor of serious diseases such as type 2 diabetes, cardiovascular disease, cancer and others. Growing evidence suggests that exposures may contribute to the progression of chronic metabolic diseases via inflammation, oxidative stress, and altered cell signaling (1–3). Additionally, epidemiological studies have suggested that individuals suffering from MetS are increasingly susceptible to particulate matter (PM)-related health effects following inhalation exposures (4). Specifically, following long-term exposure to PM_10_, individuals with MetS demonstrated elevations in circulating white blood cell counts, a non-specific hematologic marker of inflammation, compared to healthy individuals (4). The severity of PM health effects is dependent on particle size, with smaller diameter particles inducing enhanced toxicity due to deeper deposition within the lung (5–7). Epidemiological studies have demonstrated that components of MetS such as obesity and hypertension are associated with increases in markers of systemic inflammation, including white blood cell counts, C-reactive protein, and interleukin-6 following inhalation of PM_2_._5_ (8). These studies suggest that individuals with underlying disease states such as MetS may be increasingly susceptible to the inflammatory effects of inhaled particulate exposures.

Nanoparticles (NPs) represent an emerging exposure and potential health hazard due to their extremely small size and ability to evade lung clearance mechanisms. NPs have a variety of uses in numerous technologies including manufacturing procedures, electronics, consumer products, and biomedical applications. Silver NPs (AgNPs) are one of the most frequently utilized NPs and have been the focus of a number of toxicity assessments (9). Specifically, rodent pulmonary exposure to AgNPs has been determined to cause pulmonary fibrosis, and impaired pulmonary function (10, 11). These toxicity responses are related to AgNP-induced inflammatory responses and oxidative stress (12, 13). Although, individuals with pre-existing conditions such as MetS are increasingly common, the majority of NP toxicity assessment has been performed in healthy models. Individuals with MetS exist in a state of chronic inflammation that may exacerbate their responses to inhalable exposures such as NPs (8, 14, 15). MetS is associated with a dysregulation of lipids, including those involved in regulation of the immune system. Lipid mediators of inflammation are known to be modified in MetS contributing to the chronic inflammatory state associated with the condition (16). Exposure-induced inflammatory signaling is a coordinated process mediated by initial increases in lipid mediators of inflammation facilitating the acute inflammatory signaling process. This pro-inflammatory state is resolved via induction of pro-resolving lipid mediators. If not properly resolved, chronic inflammation can occur resulting in disease progression via tissue remodeling and loss of organ function (16–20). Recently, the pulmonary and systemic inflammatory response following exposure to ozone was determined to be reduced when mice were supplemented with a combination of lipid mediators of inflammatory resolution (LMIR). This demonstrates that modifications in specific lipids can influence pulmonary responses to exposures. It is likely that the lipid dysregulation involved in many diseases, such as MetS, may mediate susceptibility to exposures.

Conditions associated with dysregulation of lipids, such as high cholesterol and cardiovascular diseases are often treated with drugs to modulate circulating lipid levels. Statins are the most common therapeutic approach utilized to reduce elevated low-density lipoprotein in individuals who are at risk of cardiovascular disease (21). Research has demonstrated that statins may also exhibit anti-inflammatory properties, resulting in a variety of potential therapeutic applications, including respiratory disease treatment (22–25). Currently, there is a gap in our understanding regarding how statin treatment may impact lipid mediators of inflammation following NP exposures in MetS.

Epidemiology suggests that individuals with MetS are increasingly sensitive to air pollution exposures; however, the mechanisms remain unknown. In this study we hypothesized that MetS increases pulmonary disease susceptibility to inhaled NPs due to enhanced acute inflammatory response associated with dysregulation of lipid signaling. Elucidation of disease-related alterations in inflammatory signaling through changes in lipid metabolism are necessary to understand mechanisms of pulmonary toxicity and susceptibility. To address this problem, a MetS mouse model and a statin therapy, atorvastatin, were utilized to modulate lipids. Mice were exposed to AgNPs via oropharyngeal aspiration and the acute inflammatory response evaluated. Overall findings from this study will assist in our understanding of mechanisms of susceptibility in a prevalent subpopulation to an emerging exposure.

## Materials and Methods

### AgNP Characterization

Twenty nm citrate coated silver nanoparticles (AgNPs) were purchased from NanoComposix (San Diego, CA, United States). AgNPs were characterized to verify manufacturer’s specifications. The hydrodynamic size, polydispersion index, and ζ-potential (ZetaSizer Nano, Malvern) were assessed in DI water with AgNPs at a concentration of 25 μg/mL (*n* = 3) (Table 1). The number of AgNPs per μg was determined via NP tracking software (Nanosight, Malvern, Westborough, MA, United States) (*n* = 3) (Table 1).

**TABLE 1:** AgNP characterization.

Hydrodynamic size (nm)	Polydispersity index	ζ -potential (mV)	NanoSight (NPs/μg)
133.5 ± 38.8	0.15 ± 0.01	−44.5 ± 4.1	2.1 × 10^9^ ± 0.5

### Animals Models, Diet-Induced Metabolic Syndrome, Statin Treatment, and AgNP Exposure

C57BL/6J male mice were obtained from Jackson Labs (Bar Harbor, ME, United States) at 6 weeks of age. Mice were randomly assigned to two main groups receiving either a healthy control diet with 10% of kcal coming from fat containing 51.6 mg/kg cholesterol (D12450B, Research Diets Inc., New Brunswick, NJ, United States), or a high-fat western diet (HFWD) with 60% of kcal coming from fat containing 279.6 mg/kg cholesterol (D12492, Research Diets) for 7 weeks. This HFWD has been previously utilized and is well established to produce mouse models of MetS (26, 27). After 7 weeks, a subset of animals were transitioned to diets (healthy control or HFWD) supplemented with atorvastatin (Lipitor, Pfizer Inc., New York, NY, United States) at 10 mg/kg body weight per day (compounded by Research Diets, New Brunswick, NJ, United States) for an additional 7 weeks, while others continued on the same diet without atorvastatin. The amount of atorvastatin was matched in each diet based on calories, specifically 1.42 g of atorvastatin was incorporated per 4,057 kcals. The food was refreshed every other day and mice were weighed once a week to track weight gain over time. This resulted in four groups (1) Healthy (mice receiving the healthy diet for 14 weeks); (2) MetS (mice receiving the HFWD for 14 weeks); (3) Healthy/Statin (mice receiving the healthy diet for 14 weeks but supplemented with atorvastatin for the last 7 weeks); and (4) MetS/Statin (mice receiving the HFWD for 14 weeks but supplemented with atorvastatin for the last 7 weeks).

Following completion of 14 weeks on the diets as described above, mice were anesthetized with isoflurane and exposed to either 50 μl of phosphate buffered saline as a control or 50 μl of AgNPs at a concentration of 1 mg/ml (50 μg of AgNPs). This experimental design resulted in eight final groups: (1) Healthy-control, (2) Healthy-AgNPs, (3) MetS-control, (4) MetS-AgNPs, (5) Healthy/Statin-control, (6) Healthy/Statin-AgNPs, (7) MetS/Statin-control, and (8) MetS/Statin-AgNPs. Mice were necropsied 24 h following exposure and samples were isolated for assessment of acute toxicity. The AgNP exposure of 50 μg/mouse was selected based on previous studies demonstrating that it would stimulate an acute inflammatory response that could be utilized to examine variations due to MetS and/or statin therapy (28–30). Specifically, 50 μg of AgNPs has been shown to induce pulmonary neutrophilic recruitment and enhanced mRNA expression of markers of inflammation (29, 30). All animal procedures were conducted in accordance with the National Institutes of Health guidelines and approved by the Purdue University Animal Care and Use Committee.

### Model Characterization

At necropsy, blood was collected via cardiac puncture, serum was isolated via centrifugation, and used to evaluate circulating lipid levels. Specifically, triglycerides (Cayman Chemical, Ann Arbor, MI, United States), total cholesterol, high-density lipoprotein (HDL), and low-density lipoprotein (LDL) (Bioassay Systems, Hayward, CA, United States) were quantified utilizing commercially available kits via manufacturer’s protocols.

### Isolation of Bronchoalveolar Lavage Fluid and Cell Differential Counts

*In situ*, bronchoalveolar lavage fluid (BALF) was collected immediately after euthanasia from the right lung lobes (31). Briefly, the thoracic cavity was partly dissected, the left bronchi was tied off, and the trachea was cannulated with an 20-gauge sterile i.v. catheter. The right lung was gently lavaged by four individual aliquots of cold PBS, each at a volume of 17.5 ml/kg body weight. The first wash was placed in a 1.5 ml tube and utilized for evaluation of proteins whereas the other three were combined in a 15 ml tube. Isolated BALF was immediately centrifuged (1,800 rpm, 6 min, 4°C) to a pellet. The supernatant from the first wash, representing the protein-rich BALF fraction, was collected and stored for analysis of total protein levels using a Pierce BCA Protein-Assay Kit (Thermo Scientific, Hercules, CA, United States), albumin using Mouse Albumin ELISA Kit (ICL, Portland, OR, United States), and assessments of chemokine and cytokine levels via ELISAs (described below). Supernatant from the last three washes was discarded. BALF cell pellets from all four washes were then resuspended, combined, and counted using a cellometer. A Cytospin IV (Shandon Scientific Ltd., Cheshire, United Kingdom) was utilized to adhere an equal number of cells to microscope slides prior to staining with a three-step hematology stain (Fisher Scientific, Newington, NH, United States). Slides were viewed under a bright-filed microscope and differential cell counts were determined by examination of cellular morphology and assessment of 300 cells per slide. These counts were performed blindly by two individuals and the results averaged. The counts produced were then used to make percentages of specific cell types identified. This percentage was then applied to the sample’s total cell count number to determine the number of each cell type within each BALF sample. These assessments of the BALF are highly utilized and known to generate relevant data for the assessment of acute lung inflammation and the alveolar capillary barrier (32, 33).

### ELISA Assays to Evaluate BALF Cytokine Levels

Chemokine and cytokine levels including monocyte chemoattractant protein-1 (MCP-1), chemokine ligand 1 (CXCL1), macrophage inflammatory protein 2 (MIP-2), and interleukin-6 (IL-6) were quantified from collected BALF utilizing the Mouse DuoSet ELISA kits (R&D Systems, Minneapolis, MN, United States), according to manufacturer’s instructions.

### mRNA Expression Analysis

Total RNA was extracted from a section of the left lung lobe using Trizol (Invitrogen, Carlsbad, CA, United States) and two runs of a Bead Mill 4 homogenizer (Fisher Scientific, Newington, NH, United States) at a speed of 5 m/s (500 Watts) for 30 s. Afterward, total RNA was extracted and purified utilizing Direct-zolTM RNA MiniPrep Kits (Zymo Research) as per the manufacturer’s instructions. RNA concentration was quantified and quality assessed using a Nanodrop (Thermo Scientific, Hercules, CA, United States). An aliquot of 1 μg of RNA was reverse transcribed into cDNA using an iScript^TM^ cDNA Synthesis Kit (Bio-Rad, Hercules, CA, United States) as per the manufacturer’s instructions. Quantitative real time RT-PCR analysis was performed using inventoried primers (Qiagen, Hilden, Germany) to evaluate altered gene expression of *interleukin-6* (*IL-6*), *interleukin 1*β (*IL-1*β), *monocyte chemoattractant protein-1* (*MCP-1*), *macrophage inflammatory protein-2* (*MIP-2*), *chemokine ligand 1 (CXCL1), arachidonate 5-lipoxygenase (ALOX-5), arachidonate 15-lipoxygenase (ALOX-15)*, and *inducible nitric oxide synthase (iNOS).* In all cases, *glyceraldehyde 3-phosphate dehydrogenase (GAPDH)* was used as an internal control. Fold changes were calculated comparing all sample values individually to the average of the unexposed control healthy mouse model.

### Lipid Profiling

Multiple reaction monitoring (MRM)-profiling was used as the exploratory lipidomic approach to screen for AgNP-induced alterations in lipid mediators of inflammation, ceramides, phosphatidylcholines, and sphingomyelins (34, 35). Differential inflammation is the focus of the current investigation; therefore, we have chosen to emphasize specific lipids and pathways associated with inflammation. Lipid extraction was performed following an adapted protocol from Gouveia-Figueira et al. 2017 (36). Briefly, approximately 10 mg of the left lung was placed in 1 ml of DDI water and homogenized using three runs of a Bead Mill 4 homogenizer (Fisher Scientific, Newington, NH, United States) at a speed of 5 m/s (500 Watts) for 30 s. Then, 1 mL of ethyl acetate containing 0.1% formic acid was added to each sample and vortexed. Samples were then centrifuged at (14,000 rpm, 8 min, 25°C) to remove debris. The supernatant was transferred into a SPE Oasis Prime HLB (1CC) column and then centrifuged at (4,000 rpm, 4 min, 25°C). The columns were washed with 1 ml of 5% MeOH, and eluted with 1 mL of 100% acetonitrile (ACN). Afterward, the samples were dried in a centrifugal evaporator, vacuum-sealed, and stored at −80°C until MS analysis. The lipid extracts were reconstituted in 40 μl of 1:3 chloroform:methanol, then diluted in ACN + MeOH + 300 mM ammonium acetate (NH_4_Ac) 3:6.65:0.35 (v/v) and flow-injected (8 μL) into the ESI source of an Agilent 6410 QQQ mass spectrometer (Agilent Technologies, Santa Clara, CA, United States) operated in the negative ion mode using a micro-autosampler (G1377A). The capillary pump connected to the autosampler operated with a flow of 20 μl per minute and a pressure of 150 bar. The system was flushed with pure methanol between each sample. After flushing a quality control sample made of a mixture of lipids was injected to verify instrument performance via assessment of chromatogram intensity and time. Additionally, blanks consisting dilution solvent were injected and analyzed. Raw MS data from lung samples were processed using an in-house script and lists containing MRM transitions and the respective ion intensity values were exported to Microsoft Excel. Absolute ion intensities of all monitored transitions from samples were compared to blank samples. Transitions that yielded signal above the level seen in blank samples were selected. Results showed are tentative attributions based on flow injection and MRM and have not been validated by LC-MS/MS with the use of isotopically labeled internal standards. However, previous studies have utilized this method to comprehensively examine lipid alterations (34, 35, 37–44). Relative ion intensities were then normalized to tissue mass and statistically evaluated.

### Statistical Analyses

Results are expressed as mean values ± S.E.M. with 5–6 animals/group. All samples were assessed in duplicate and values averaged for endpoints including serum parameters, BALF cell counts, mRNA expression, and BALF chemokines and cytokines. For the MRM-profiling method, each lung sample was assessed once to determine lipid modifications. For statistical analysis, a three-way analysis of variance (ANOVA) was used to determine statistical differences between groups, with disease (healthy or MetS), exposure (control or AgNPs exposure), and treatment (no statin or statin) as the three factors. A Bonferroni test was utilized for multi-comparison analysis. All statistical examinations were performed using GraphPad Prism 8 software (Graph Pad, San Diego, CA, United States), and *p* < 0.05 was considered to be statistically significant.

## Results

### Model Characterization

**FIGURE 1:**
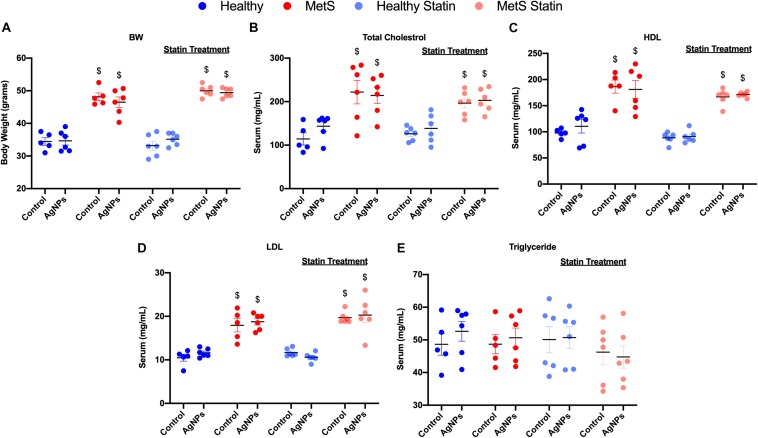
Characterization of body weight and serum lipid levels in healthy and MetS mouse models following 14 weeks on either a control or high-fat western diet (HFWD). Healthy and MetS mice without or with statin treatment were characterized by examination of **(A)** body weight, **(B)** serum total cholesterol, **(C)** serum high-density lipoprotein (HDL), **(D)** serum low-density lipoprotein (LDL), and **(E)** serum triglyceride levels following oropharyngeal aspiration of saline (control) or AgNPs (50 μg). Values are expressed as mean ± standard error of mean (SEM, *n* = 5–6). $ denotes significant differences between healthy and MetS, (*p* < 0.05).

C57B6J mice on the HFWD demonstrated increased body weight-gained and total cholesterol levels compared to mice fed a healthy control diet (Figures 1A,B). Circulating levels of HDL and low-density lipoprotein (LDL) levels were increased due to diet (Figures 1C,D). However, no changes were observed in triglyceride levels between the mouse models on differing diets (Figure 1E). Statin treatment did not alter body weight, nor did it alter circulating levels of total cholesterol, HDL, LDL, and triglycerides (Figures 1A,C–E). AgNP exposure was not determined to modify parameters characterized including body weight, total cholesterol, HDL, LDL, or triglyceride levels in any of the mouse models (Figure 1).

### Acute Inflammatory Response

**FIGURE 2:**
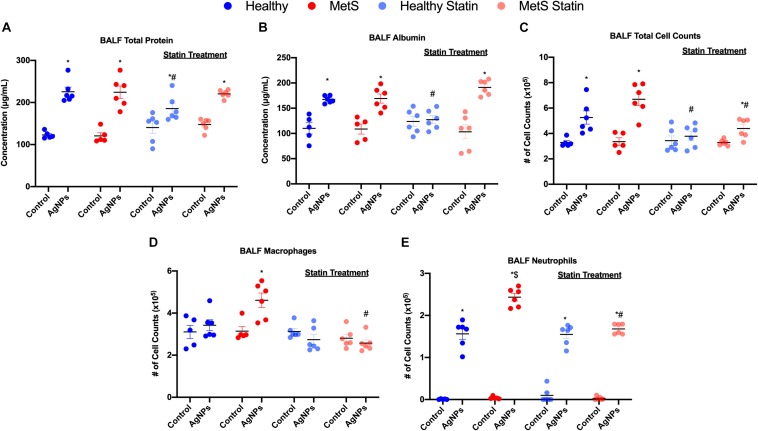
Effect of AgNP exposure on BALF **(A)** total protein, **(B)** albumin, **(C)** total cells, **(D)** macrophages and **(E)** neutrophils from healthy and MetS mice and the influence of statin treatment. Values are expressed as mean ± SEM (*n* = 5–6). * denotes significant differences due to AgNP exposure, $ denotes significant differences between healthy and MetS mouse models, # denotes significant differences due to statin treatment (*p* < 0.05).

Following oropharyngeal aspiration exposure to 50 μg AgNPs, assessment of BALF total protein levels, albumin levels, and cytometry were performed to examine exposure-induced alterations in the alveolar capillary barrier and the acute inflammatory response. Total protein concentration in BALF, a marker of pulmonary edema and alveolar capillary barrier integrity, was significantly elevated similarly in both healthy and MetS mice due to AgNP exposure in comparison to model-matched controls (Figure 2A). BALF levels of the high molecular weight protein, albumin, were significantly elevated similarly in both models due to AgNP exposure further suggesting loss of alveolar capillary barrier integrity and pulmonary edema (Figure 2B). To determine AgNP-induced acute pulmonary inflammation, alterations in BALF cells were examined. Total cell counts in the BALF were significantly elevated in both exposed healthy and MetS mice compared to model-matched controls (Figure 2C). This increase in BALF total cell counts was more prominent in MetS mice compared to healthy (Figure 2C). Following AgNP exposure, MetS mice demonstrated increased macrophage numbers within the BALF (Figure 2D). Both healthy and MetS mice demonstrated increases in BALF neutrophils following AgNP exposure (Figure 2E). The influx of neutrophils following AgNP exposure was exacerbated in the MetS mouse model compared to healthy (Figure 2E). These increases in BALF neutrophil numbers are indicative of active inflammation within the lung. Representative images of slides used for analysis of BALF cellular content are available in Supplementary Figure S1.

Statin treatment was determined to modulate many of these AgNP-induced responses within the BALF. Statin treatment did not alter BALF total protein concentration, albumin levels, total cell counts, macrophage counts, or neutrophil counts in control mice (unexposed) compared to those not receiving statins (Figure 2). Healthy mice on statins exposed to AgNPs demonstrated decreased BALF total protein levels compared to healthy mice not receiving statins (Figure 2A). MetS mice on statins demonstrated similar increases in BALF total protein levels in response to AgNP exposure as observed in MetS mice not receiving statins (Figure 2A). Statin treatment inhibited AgNP-induced increases in BALF albumin levels in healthy mice (Figure 2B). AgNP exposure resulted in increased BALF albumin levels in MetS mice receiving statins similar to changes in albumin measured in MetS not receiving statins (Figure 2B). Statin treatment inhibited increases in BALF total cell counts observed in healthy mice following AgNP exposure (Figure 2C). Although the magnitude of BALF total cell influx was reduced, significant elevations were still measured in MetS receiving statin treatment due to AgNP exposure (Figure 2C). Statin treatment inhibited AgNP-induced BALF macrophage cell influx in MetS mice (Figure 2D). Lastly, statin treatment did not change the influx of neutrophils into the BALF in AgNP-exposed healthy mice (Figure 2E). A significant decrease in AgNP-induced neutrophilic influx was observed in the MetS mouse model due to statin treatment compared to those not receiving statins (Figure 2E).

### Pulmonary mRNA Gene Expression

**FIGURE 3:**
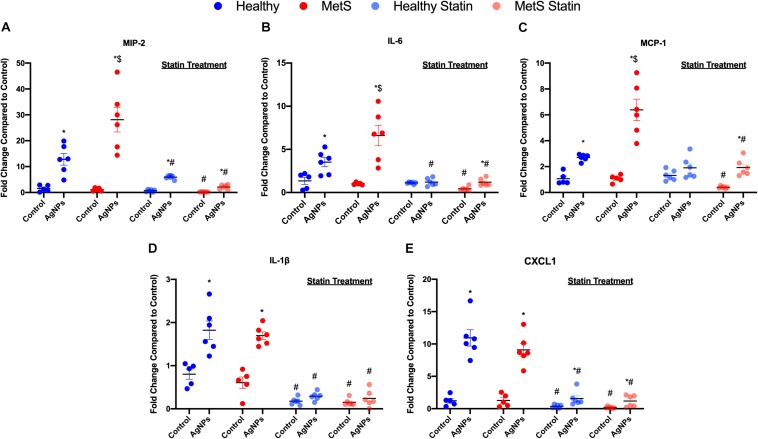
Effect of AgNP exposure on pro-inflammatory gene expression in lung tissue from healthy or MetS mice and the influence of statin treatment. AgNP-induced alterations in the gene expression of **(A)** *macrophage inflammatory protein-2* (*MIP-2*), **(B)** *interleukin-6* (*IL-6*), **(C)** *monocyte chemoattractant protein-1* (*MCP-1*), **(D)** *interleukin-1*β (*IL-1*β), and **(E)** *chemokine 1* (*CXCL1*) were evaluated in lung tissue. Values are expressed as mean ± SEM (*n* = 5–6). * denotes significant differences due to AgNP exposure, $ denotes significant differences between healthy and MetS mouse models, # denotes significant differences due to statin treatment (*p* < 0.05).

Pro-inflammatory mRNA gene expression levels in lung tissue samples were examined to determine differential induction due to MetS following AgNP exposure. *Macrophage inflammatory protein-2* (*MIP-2*), *monocyte chemoattractant protein-1* (*MCP-1*), *interleukin-6* (*IL-6*), *interleukin-1*β *(IL-1*β*)*, and *chemokine ligand 1 (CXCL1)* mRNA levels were significantly elevated in both healthy and MetS models following AgNP exposure compared to model-matched controls (Figure 3). Following AgNP exposure, MetS mice demonstrated exacerbated induction of *MIP-2*, *IL-6*, and *MCP-1* compared to healthy mice (Figures 3A–C). Induction of *IL-1*β and *CXCL1* was similar in response to AgNP exposure between both healthy and MetS mouse models (Figures 3D,E). Statin treatment significantly decreased the pro-inflammatory mRNA gene expression levels observed in both models following AgNP exposure (Figure 3). Although reduced, healthy mice receiving statins still demonstrated induction of *MCP-1*, and *CXCL1* following AgNP exposure (Figures 3C–E) while MetS mice receiving statins still exhibited induction of *MIP-2*, IL-6, *MCP-1*, and *CXCL1* (Figures 3A,C,E).

**FIGURE 4:**
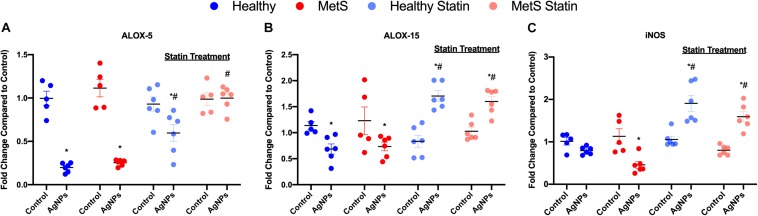
Alterations in genes involved in lipid metabolism and signaling following AgNP exposure in lung tissue from healthy and MetS mice and the influence of statins. AgNP-induced alterations in the gene expression of **(A)** *arachidonate-5 lipoxygenase* (*ALOX-5*), **(B)** *arachidonate 15-lipoxygenase* (*ALOX-15*), and **(C)** *inducible nitric oxide synthase* (*iNOS*) were evaluated in lung tissue. Values are expressed as mean ± SEM (*n* = 5–6). * denotes significant differences due to AgNP exposure, and # denotes significant differences due to statin treatment (*p* < 0.05).

To evaluate mediators of lipid metabolism within the lung, alterations in gene expression of the lipoxygenases *arachidonate-5 lipoxygenase* (*ALOX-5*), and *arachidonate 15-lipoxygenase* (*ALOX-15*) were examined (Figures 4A,B). AgNP exposure reduced *ALOX-5* and *ALOX-15* mRNA expression in both healthy and MetS mouse models (Figures 4A,B). Although *ALOX-5* was still significantly decreased due to AgNP exposure, statin treatment inhibited the magnitude of decrease observed in the healthy mouse model (Figure 4A). Statin treatment was found to completely inhibit changes observed in *ALOX-5* in the MetS model following AgNP exposure (Figure 4A). Additionally, both healthy and MetS mice receiving statin treatment demonstrated significantly increases in *ALOX-15* levels following AgNP exposure (Figure 4B). *ALOX-5* and *ALOX-15* produce lipid signaling mediators that are known to up-regulate *inducible nitric oxide synthase (iNOS).* AgNP exposure was determined to reduce *iNOS* gene expression in only the MetS mice (Figure 4C). Statin treatment resulted in upregulation of *iNOS* gene expression following AgNP exposure in both healthy and MetS mice (Figure 4C).

### Inflammatory Cytokines

**FIGURE 5:**
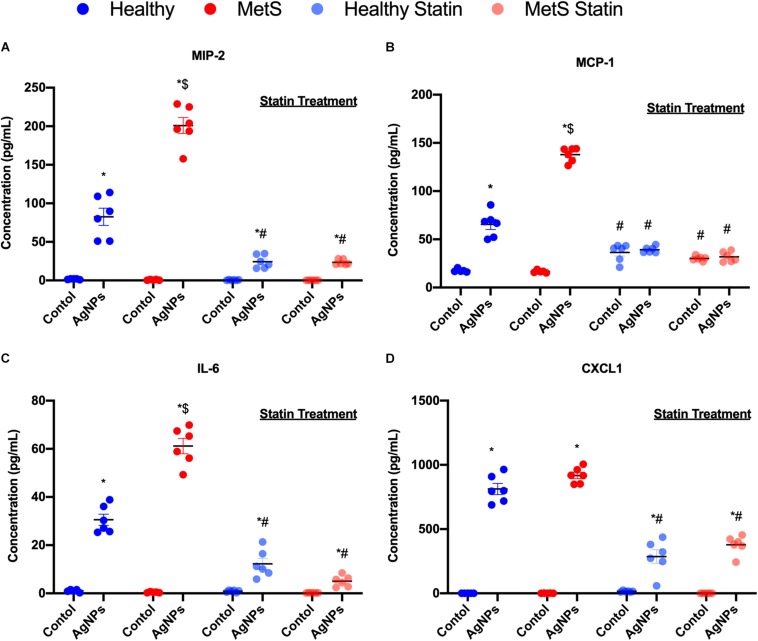
Alterations in BALF cytokine/chemokines proteins due to AgNP exposure in healthy and MetS mice and the effect of statin treatment. Proteins levels of **(A)** MIP-2, **(B)** MCP-1, **(C)** IL-6, and **(D)** CXCL1 protein levels were examined in BALF to determine disease-related differences in AgNP-induced inflammatory response. Values are expressed as mean ± SEM (*n* = 6–8). * denotes significant differences due to AgNP exposure, $ denotes significant differences between healthy and MetS mouse models, # denotes significant differences due to statin treatment (*p* < 0.05).

Inflammatory cytokine protein levels were examined within the BALF to determine alterations in pulmonary inflammation response to AgNP exposure between healthy and MetS mice (Figure 5). MIP-2, MCP-1, IL-6, and CXCL1 were elevated in both healthy and MetS mice following AgNP exposure compared to model-matched controls (Figure 5). MetS mice exposed to AgNPs demonstrated enhanced BALF levels of MIP-2, MCP-1, and IL-6 compared to exposed healthy mice (Figures 5A–C). CXCL1 protein levels were induced similarly in both animal models following AgNP exposure (Figure 5D). Statin treatment was determined to inhibit AgNP-induced increases in all inflammatory proteins (Figure 5). Although still elevated compared to unexposed controls receiving statins, BALF MIP-2, IL-6, CXCL1 were significantly reduced compared to mice not receiving statins (Figures 5A,C,D). In both healthy and MetS mice, statin treatment inhibited AgNP-induced BALF MCP-1 levels to those observed in unexposed controls treated with statins (Figure 5B).

### Lipid Profiles

An exploratory lipidomic approach, MRM-profiling, was used to screen AgNP-induced alterations in pulmonary lipids due to MetS. MRM-profiling was originally proposed as a two-phase (discovery and screening) method based on flow injection and chemical functional profiling for small molecule biomarker discovery (34, 45). It has been applied to various studies (37–42) and here, we used the approach to interrogate the samples for MRMs related to lipid mediators reported in the literature (46–49). The ion intensities yielded were compared to a blank in order to eliminate the ones that were not informative. The remaining data were evaluated and interpreted in combination with the other data reported to build molecular mechanistic hypotheses.

**FIGURE 6:**
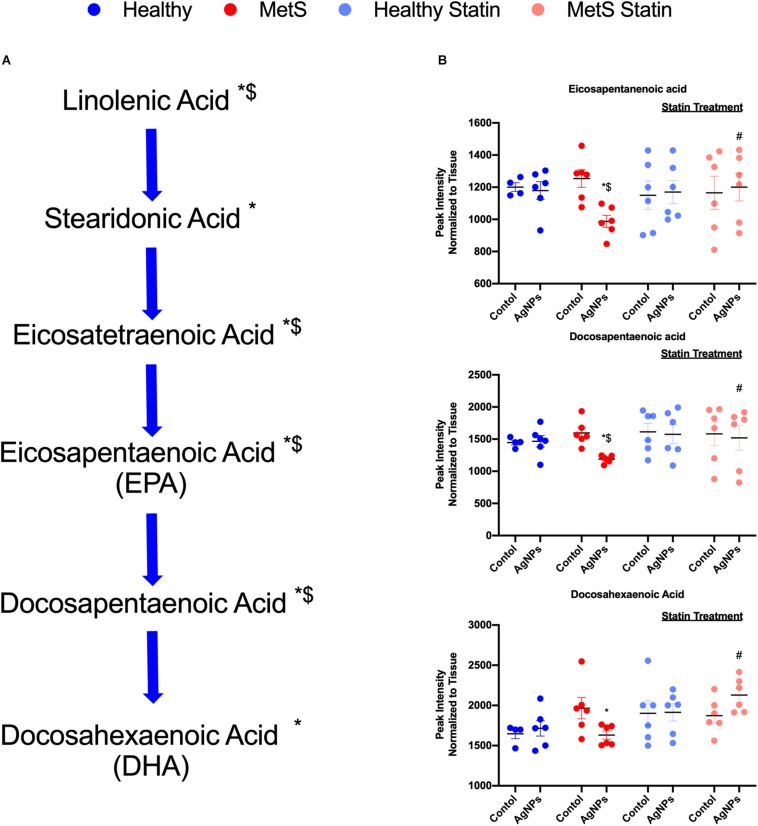
Alterations in lipid mediators of inflammation resolution (LMIR) in the lungs of healthy and MetS mice following AgNP exposure and the effect of statins. Exploratory lipid profiling suggests variations in lipids involved in the synthesis of **(A)** eicosapentaenoic acid (EPA) and docosapentaenoic acid (DHA). * denotes lipids significantly reduced only in MetS in response to AgNP exposure, while $ denotes lipids significantly reduced in AgNP exposed MetS compared to AgNP exposed healthy mice (*p* < 0.05). **(B)** Representative lipids within the EPA and DHA pathway altered due to AgNP in healthy and MetS mice. * denotes lipids significantly reduced only in MetS in response to AgNP exposure, while $ denotes lipids significantly reduced in exposed MetS compared to exposed healthy mice, and # denotes significant differences due to statin treatment (*p* < 0.05). Graphs of other components of the pathway can be found in Supplementary Figure S2.

**FIGURE 7:**
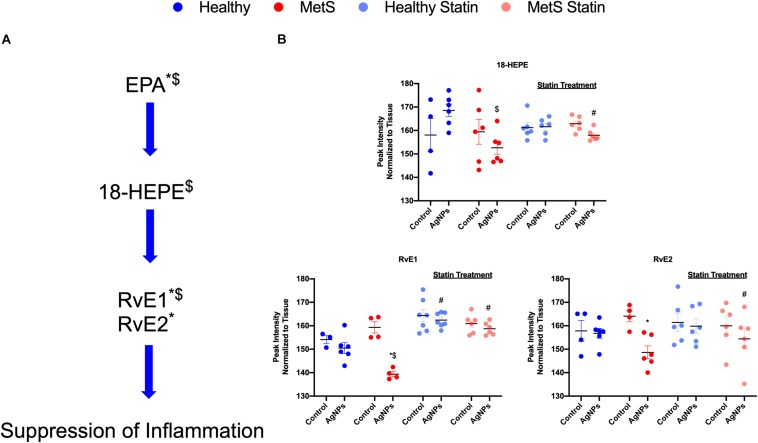
Assessment of eicosapentaenoic acid (EPA)-derived lipid mediators of inflammatory resolution (LMIR) in the lungs of healthy and MetS mice following AgNP exposure and the effect of statins. **(A)** EPA is metabolized to resolvin E1 (RvE1) and E2 (RvE2) via the intermediate 18-HEPE. * denotes lipids significantly reduced only in MetS in response to AgNP exposure, while $ denotes lipids significantly reduced in exposed MetS compared to exposed healthy mice (*p* < 0.05). **(B)** Quantitative differences in EPA-derived LMIR due to AgNP exposure in MetS and healthy mice and the influence of statin treatment. * denotes lipids significantly reduced only in MetS in response to AgNP exposure, while $ denotes lipids significantly reduced in exposed MetS compared to exposed healthy mice, and # denotes significant differences due to statin treatment (*p* < 0.05).

**FIGURE 8:**
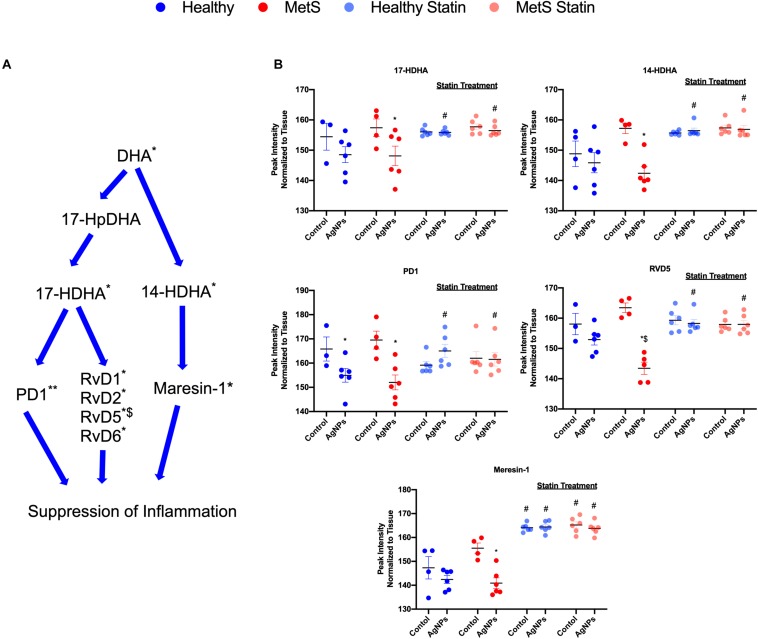
Assessment of docosahexaenoic acid (DHA)-derived LMIR in the lung tissue of healthy and MetS mice following AgNP exposure and the effect of statins. **(A)** DHA is metabolized to protectin D1 (PD1) and the resolvin D series (RvD1, RvD2, RvD5, and RvD6) via intermediates 17-HpDHA and 17-HDHA. Additionally, DHA can be metabolized to maresin-1 via 14-HDHA. * denotes lipids significantly reduced in MetS in response to AgNP exposure, ** denotes lipids significantly reduced in both healthy and MetS mice in response to AgNP exposure, $ denotes lipids significantly reduced in exposed MetS mice compared to exposed healthy mice (*p* < 0.05). **(B)** Quantitative differences in representative DHA-derived LMIR due to AgNP exposure in MetS and healthy mice and the influence of statin treatment. * denotes lipids significantly reduced in MetS in response to AgNP exposure, $ denotes lipids significantly reduced in exposed MetS mice compared to exposed healthy mice, and # denotes significant differences due to statin treatment (*p* < 0.05). Graphs of other components of the pathway can be found in Supplementary Figure S3.

Alterations were observed for lipid mediators of inflammatory resolution (LMIR) within the isolated lung tissue (Figure 6). Specifically, MetS mice exposed to AgNPs demonstrated reduced levels of linolenic acid, stearidonic acid, eicosatetraenoic acid, eicosapentaenoic acid (EPA), docosapentaenoic acid, and docosahexaenoic acid (DHA) (Figures 6A,B and Supplementary Figure S2). Alterations of this pathway were not observed in the healthy mouse model following AgNP exposure (Figure 6B). Statin treatment was observed to inhibit reductions in all components of this pathway when compared to AgNP exposed MetS mice not receiving statins (Figure 6B). Additionally, reductions were observed in LMIR produced via metabolism of EPA following AgNP exposure only in MetS mice (Figure 7). These included decreased levels of the intermediate 18-HEPE, as well as resolvin E1 (RvE1), and E2 (RvE2), which signal for suppression of inflammation (Figure 7A). Statin treatment was determined to inhibit these AgNP-induced reductions in EPA-derived LMIR observed in the MetS mouse model not receiving statin treatment (Figure 7B). DHA-derived LMIR were also determined to be modified primarily in the MetS mouse model following AgNP exposure (Figure 8 and Supplementary Figure S3). DHA can be metabolized into 17-HpDHA, and then to 17-HDHA which produces protectin D1 (PD1) or the resolvin D series (RvD1, RvD2, RvD5, and RvD6) (Figure 8A). Further, DHA can be metabolized into 14-HDHA and then to maresin-1 (Figure 8A). The LMIRs produced via DHA metabolism suppress inflammation and reestablish homeostasis. All components of this pathway were determined to be reduced in response to AgNP exposure in MetS mice (Figure 8A). Only PD1 was found to be reduced in healthy mice following AgNP exposure (Figures 8A,B). Statin treatment was observed to inhibit AgNP-induced reductions in LMIR observed the MetS mouse model not receiving statin treatment (Figure 8B and Supplementary Figure S3). Further, statin treatment also inhibited PD1-decreases observed in healthy mice not receiving statin treatment (Figure 8B and Supplementary Figure S3).

## Discussion

Underlying disease states are increasingly prevalent and may represent susceptible subpopulations that need to be taken into account within toxicity assessments of emerging exposures. Individuals suffering from MetS have demonstrated enhanced inflammation following inhalation exposure to particulate matter, though the mechanisms responsible are still not fully understood (50, 51). In this study, we hypothesized that a mouse model of MetS would be increasingly sensitive to AgNP-induced acute inflammation compared to a healthy mouse model due to disease-associated dyslipidemia. Our findings demonstrate an enhanced acute pulmonary inflammatory response in MetS mice compared to healthy. This exacerbated response in the MetS mouse model was associated with decreased gene expression of enzymes involved in lipid metabolism as well as reductions in LMIR. The use of atorvastatin was determined to inhibit AgNP-induced acute pulmonary inflammation in the MetS model. Further, statin treatment inhibited alterations in the relative ion intensities of LMIR in the MetS mouse model following AgNP exposure.

Nanoparticle toxicity is related to their size, which allows for deeper deposition within the lung and evasion of typical mechanisms which clear larger particulates from the lung. Similar to our findings, other studies have demonstrated that pulmonary exposure to AgNPs induces acute inflammation characterized by neutrophilic influx and modifications in inflammatory mediators (52–54). However, these studies have focused primarily on healthy animal models and have not investigated pulmonary responses due to NP exposure in MetS. Investigations using models of obesity, a component of MetS, have demonstrated exacerbated ozone-induced pulmonary effects, including airway hyperresponsiveness, neutrophil recruitment, and inflammatory signaling compared to non-obese mice (55–57). Our study determined that mice with MetS exhibited an exacerbated inflammatory response compared to healthy in response to AgNP exposure. Specifically, increased neutrophilic influx occurred that corresponded with enhanced transcription and translation of distinct inflammatory cytokines and chemokines. This enhanced recruitment of neutrophils into the lungs is most likely mediated by increased expression of *MIP-2*, *IL-6*, and *MCP-1*, observed in MetS mice compared to healthy mice in response to AgNP exposure. Further, IL-1β and CXCL1 likely do not account for the exacerbated neutrophil recruitment in MetS mice, since their levels were observed to be similar between both models in response to AgNP exposure. Together, this suggests that MetS exacerbates inflammatory signaling via induction of specific chemokines and cytokines including MIP-2, IL-6, and MCP-1. Previously, it has been demonstrated that increases in *MCP-1* may suppress *iNOS*, thereby enhancing lung inflammation (58). Further, *iNOS* expression by macrophages also has been linked to the suppression of IL-1β and a reduction in M1 macrophages (59, 60). This suggests that the reductions in *iNOS* gene expression observed in MetS mice following AgNP exposure may contribute to the exacerbated influx of neutrophils observed. Many exposures are known to modulate iNOS activity, suggesting that iNOS dysregulation may contribute to MetS exacerbated responses to a number of exposures. The contribution of iNOS dysregulation and nitric oxide to inflammatory responses in susceptible models requires additional investigation.

Lipids are intricately involved in the inflammatory process and their dysregulation is known to be a key component in MetS. Our exploratory profile data indicates that AgNP exposure resulted in decreased levels of LMIR produced via metabolism of eicosapentaenoic acid (EPA) and docosahexaenoic acid (DHA) solely in the MetS mouse model. A previous investigation demonstrated that ozone exposure reduces lung levels of these DHA and EPA-derived LMIR in healthy mice (61). Further, supplementation of mice via intraperitoneal injection of a combination of LMIRs was observed to protect them from ozone-induced inflammation and lung damage (61). Healthy mice in our study did not demonstrate alterations in many of these lipids at the time point evaluated suggesting that MetS mice may be increasingly susceptible to exposures via the dysregulation of specific lipids. These DHA and EPA-derived lipids regulate the immune system by promoting a return to homeostasis following an inflammatory event. For example, resolvins inhibit proinflammatory signals such as IL-6, MCP-1, and others (62). Additionally, LMIRs such as RvD2 and others have been shown to decrease neutrophil adhesion to the endothelium and tissue recruitment via induction of iNOS (63, 64). Exposure-induced reductions in resolvins and other LMIR may be responsible for exacerbated pro-inflammatory signaling observed due to MetS. Overall, only minor quantitative alterations were observed for each LMIR due to AgNP exposure in MetS mice. However, there is likely an additive effect occurring due to all assessed metabolites and LMIRs trending toward downregulation. Investigation of a single time point also limits our capacity to examine metabolic pathways where alterations may be more robust at earlier time points or may be sustained for longer periods of time. Inflammation is known to be a common factor of many MetS components (obesity, insulin resistance, etc.) (65) and is a contributing factor to progression of MetS to more serious conditions such as diabetes, cardiovascular disease, and others. These exacerbated inflammatory responses observed in MetS mice due to AgNP exposure-induced lipid dysregulation may advance the progression of these disease conditions.

Lipoxygenases are involved in the metabolism of DHA and EPA to their immune active components. Our study demonstrated that *arachidonate 5-lipoxygenase (ALOX-5)* and *arachidonate 15-lipoxygenase (ALOX-15)* were transcriptionally down-regulated in both healthy and MetS mouse models due to AgNP exposure. Ambient PM_2_._5_ exposure has been demonstrated in lung epithelial cells to inhibit ALOX-15, leading to enhances in markers of tumorigenesis though the mechanisms for this inhibition are unknown (66). Further, inhalation exposure of ozone also reduces ALOX-15 protein levels within the lung (61). Both our data and findings by other researchers suggest that the modifications in LMIR observed within our study may be due to inhibition of lipoxygenases within the lung of MetS mice. Future studies are necessary to determine distinct mechanisms responsible for ALOX inhibition caused by exposures and why MetS may enhance these responses. Although, previous studies have demonstrated the utilization of the MRM profiling method, additional future studies are needed to confirm exploratory lipid profiling results via LC-MS/MS (35, 37–44). Our current study only evaluated transcriptional regulation of ALOX, future studies are needed to examine protein levels and activity. Interestingly, lipids involved in the metabolism of α-linolenic acid to EPA and DHA were also determined to be uniquely decreased in the MetS mouse model due to AgNP exposure. These results suggest that the endogenous production EPA and DHA may also be reduced in MetS further diminishing LMIR levels in response to exposure. Overall, this disruption in LMIR may result in chronic inflammation and a delay in the return to homeostasis.

Together our findings support that MetS-associated dyslipidemia may enhance susceptibility to AgNP exposure via modulations in LMIR. Statins represent a common therapy for dyslipidemia which are highly utilized in the MetS subpopulation (67). Statins lower cholesterol, decreasing cardiovascular disease and stroke in at risk populations such as those with MetS. They reduce cholesterol biosynthesis by inhibiting 3-hydroxy-3-methylglutaryl coenzyme A reductase. In our study, 7 weeks of statin treatment was observed to slightly modify circulating lipid levels; however, the alterations were not statistically significant. This lack of a statistically difference due to statin treatment is likely due to variability in lipid levels observed within our MetS model (Figure 1). Previous investigation, also administering 10 mg/kg/day atorvastatin in the diet of mice demonstrated similar slight reductions in circulating lipids (68). Studies have also demonstrated that statins have additional beneficial responses beyond lowering cholesterol such as increased vasodilation via increasing nitric oxide production, inhibiting clot formation, and anti-inflammatory effects (69–71). Our study supports that statin treatment may also be protective, reducing inflammation that occurs in certain susceptible populations as a result of exposures. Atorvastatin has been previously been shown to increase anti-inflammatory lipids such as 15-epi-LXA4 in the heart (17, 72). Our study suggests that atorvastatin may also be able to inhibit exposure-induced changes via modulation of levels of DHA- and EPA-derived LMIR within the lung. Interestingly, statin treatment lowered neutrophilic influx in MetS mice to levels equivalent to the healthy group in response to AgNP exposure. This suggests that statin treatment may reduce the susceptibility to particulate exposures associated with MetS. Similar to our findings, statins have been shown to reduce the pulmonary and systemic inflammatory response (neutrophil influx and circulating IL-6 levels) in rabbits following PM_10_ exposure (22, 23). Systemic effects are known to occur following PM inhalation exposures such as cardiovascular toxicity (73). Statins have also been shown to reduce PM_2_._5_-induced myocardial inflammation in rats demonstrating their potential to inhibit systemic effects of inhalation exposures (55). Specifically, our study suggests that statins may reduce inflammation via modulation of enzymes which are involved in the production of LMIR, such as ALOX-5 and 15. Studies have previously demonstrated that statins can modulate cycloxygenase-2 activity influencing LMIR production (72). Together, our data suggests that statin therapy may reduce MetS-associated susceptibility to exposures by maintaining LMIR content within the lung following an exposure.

## Conclusion

**FIGURE 9:**
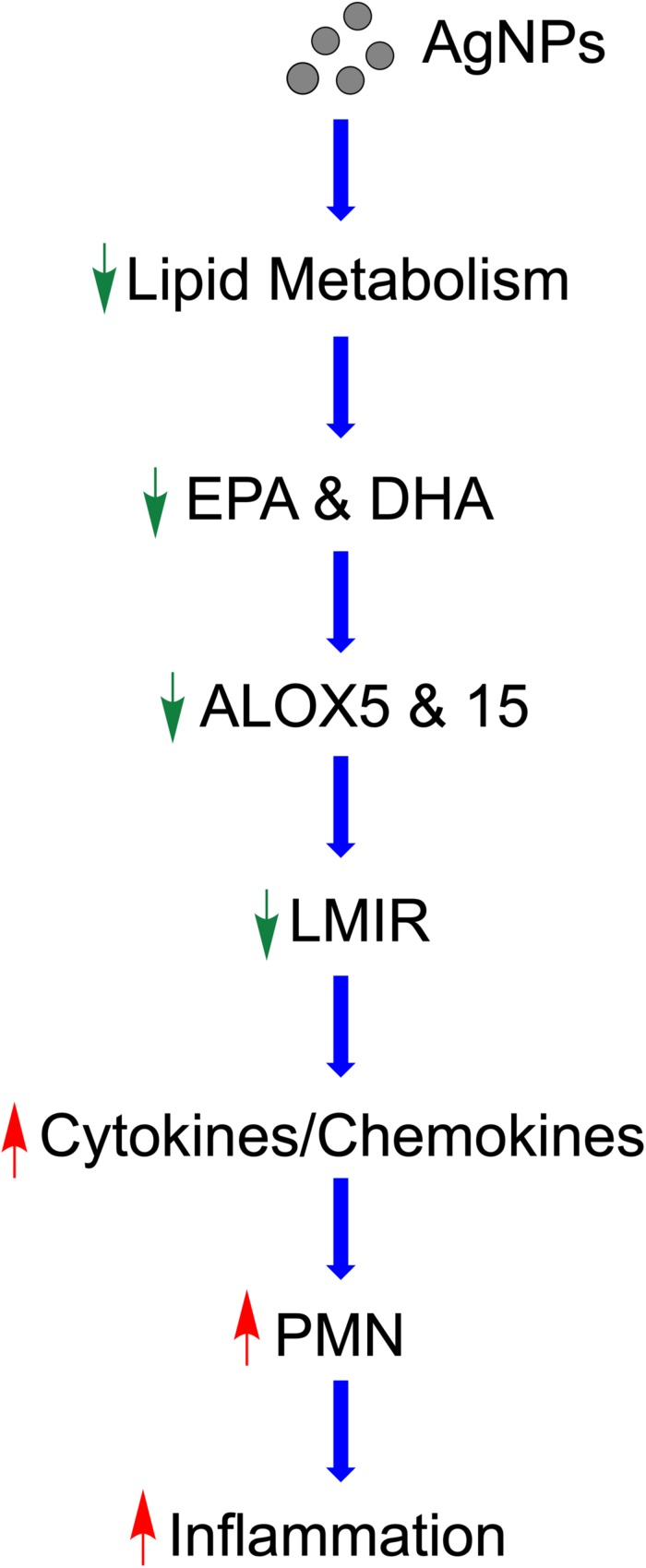
Proposed mechanism by which AgNPs induce exacerbated inflammation within the lungs of MetS mice. AgNP exposure disrupts lipid metabolism, specifically EPA and DHA. Reduced ALOX decreases production of LMIR causing decreased iNOS. This removal of inhibitory inflammatory signaling results in enhanced pro-inflammatory cytokine/chemokine levels leading to exacerbated recruitment of neutrophils into the lung and an enhanced acute inflammatory response. Statin therapy inhibits these exacerbated inflammatory responses and may allow for inflammatory signaling to occur in a similarly manner to healthy mice.

Taken together, our study demonstrates a potential mechanism by which MetS may enhance the acute pulmonary inflammatory response following nano-sized particulate exposures (Figure 9). Specifically, this pathway involves AgNP-induced alterations in lipid metabolism reducing LMIR and exacerbating pulmonary inflammatory signaling (Figure 9). Statins which are known to modulate lipid levels were determined to inhibit these AgNP-induced lipid alterations and inhibit exacerbated acute inflammatory responses observed in the MetS mouse model. These AgNP induced effects may be applicable to a number of other exposures that induce similar signaling pathways such as other forms of particulate matter. It is difficult to examine metabolic pathways at a single time point; however, our study demonstrates for the first time that MetS may enhance pulmonary susceptibility to AgNP exposure via altered lipid metabolism. Future studies utilizing additional time points are needed to evaluate earlier time points where pro-inflammatory lipids may exacerbate acute pulmonary responses to exposures in MetS. Further, it is unknown if these reductions in LMIR may be more robust at earlier time points or predispose MetS mice to chronic inflammation leading to altered disease progression. A comprehensive assessment and analytical validation of our lipidomic data is currently being performed to evaluate alterations in lipid mediators of inflammation, ceramides, phosphatidylcholines and sphingomyelins due to MetS, AgNP exposure, and statin treatment. Interventional studies are underway to determine the role of specific LMIRs in the exacerbated response observed in MetS. Additional, studies also need to be performed to determine cell-specific responses mediating these alterations in inflammation. Our data (Supplementary Figure S1) and previous investigations have demonstrated that AgNPs following inhalation are internalized via alveolar macrophages (74). Due to their presence in macrophages and their role in immune signaling, macrophages are the most likely cells within the lung mediating the differential responses observed. To effectively protect the public, it is necessary to examine prevalent diseases that may predispose individuals to exacerbated responses to exposures. Elucidation of the mechanisms underlying susceptibility can lead to the development of improved prevention and therapeutic strategies.

## Data Availability Statement

The raw data supporting the conclusions of this article will be made available by the authors to any qualified researcher on request.

## Ethics Statement

The animal study was reviewed and approved by the Purdue University Animal Care and Use Committee.

## Author Contributions

JS designed the study. SA, LK, LX, XD, and JS performed the exposures and sample collection procedures. SA, LX, and XD performed assays to evaluate inflammation and toxicity. SA, LK, JM, and CF contributed to the assessment of lipids, bioinformatics, and data analysis. SA and JS contributed to drafting the manuscript. All authors contributed to the revision and approved the final submitted manuscript.

## Conflict of Interest

The authors declare that the research was conducted in the absence of any commercial or financial relationships that could be construed as a potential conflict of interest.

## Supplementary Material

FIGURE S1 Microscopy images of cells counted from collected bronchoalveolar lavage fluid. **(A)** Healthy Control, **(B)** Healthy AgNP exposure, **(C)** MetS Control, **(D)** MetS AgNP exposure, **(E)** Healthy/Statin Control, **(F)** Healthy/Statin AgNP exposure, **(G)** MetS/Statin Control, and **(H)** MetS/Statin AgNP exposure. Representative macrophages are denoted with a green arrow and representative neutrophils are denoted with a red arrow. AgNPs can be observed as black debris within macrophages from exposed animals. The images shown are at a magnification of 20x with a bright field microscope.

FIGURE S2 Alterations in lipid mediators of inflammation resolution (LMIR) in response to AgNP exposure. Lipids are tentative attributions based on flow injection and multiple reaction monitoring. Alterations in **(A)** linolenic acid, **(B)** stearidonic acid, and **(C)** eicosatetraenoic acid were observed due to MetS in response to AgNP exposure. ^∗^ denotes lipids significantly reduced only in MetS in response to AgNP exposure, while $ denotes lipids significantly reduced in exposed MetS compared to exposed healthy mice, and # denotes significant differences due to statin treatment (*p* < 0.05).

FIGURE S3 Assessment of docosahexaenoic acid (DHA)-derived lipid mediators of inflammatory resolution (LMIR). Lipids are tentative attributions based on flow injection and multiple reaction monitoring. Alterations in **(A)** 17-HpDHA, **(B)** RvD1, **(C)** RvD2, and **(D)** RvD6 were observed due to MetS in response to AgNP exposure. ^∗^ denotes lipids significantly reduced in MetS in response to AgNP exposure, and # denotes significant differences due to statin treatment (*p* < 0.05).
