# Supplementary figures and images for: Exacerbation of Nanoparticle-Induced Acute Pulmonary Inflammation in a Mouse Model of Metabolic Syndrome
Source: Front Immunol. 2020 May 7;11:818. doi: 10.3389/fimmu.2020.00818 (PMC7221136; doi:10.3389/fimmu.2020.00818)

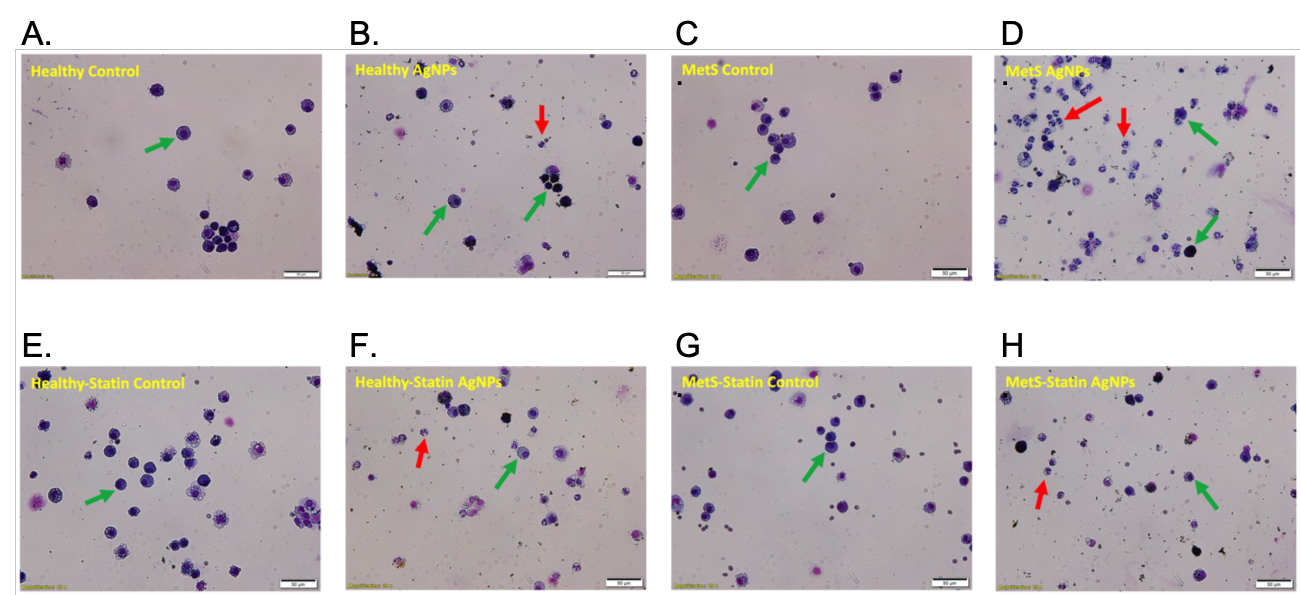

Supplement: FIGURE S1 — Microscopy images of cells counted from collected bronchoalveolar lavage fluid. (A) Healthy Control, (B) Healthy AgNP exposure, (C) MetS Control, (D) MetS AgNP exposure, (E) Healthy/Statin Control, (F) Healthy/Statin AgNP exposure, (G) MetS/Statin Control, and (H) MetS/Statin AgNP exposure. Representative macrophages are denoted with a green arrow and representative neutrophils are denoted with a red arrow. AgNPs can be observed as black debris within macrophages from exposed animals. The images shown are at a magnification of 20x with a bright field microscope. [file Image_1.tiff]

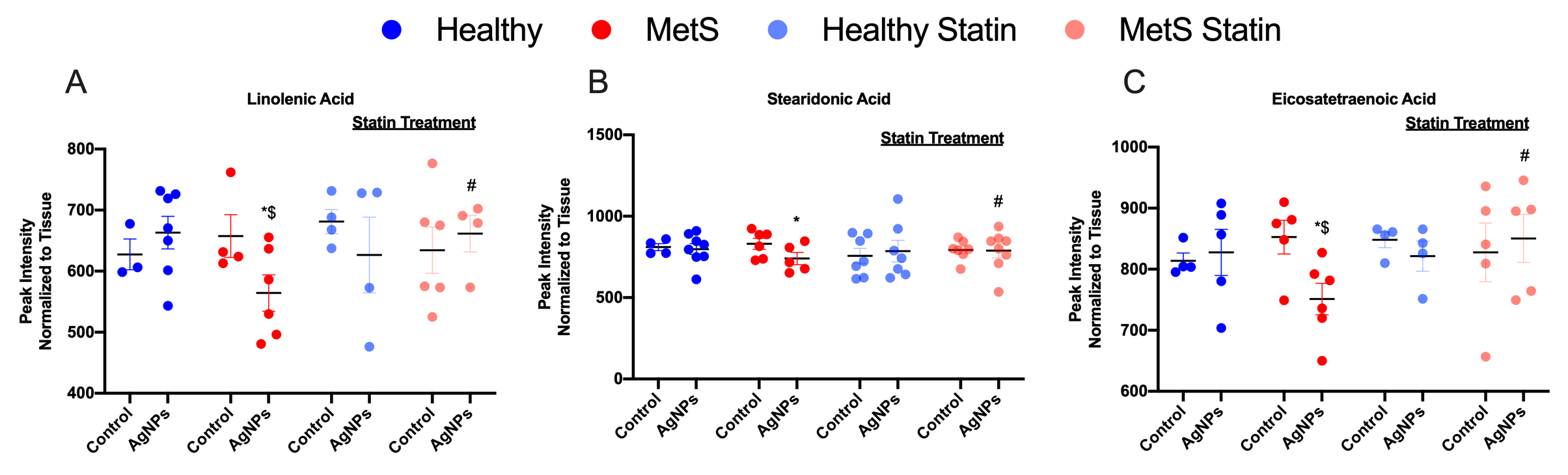

Supplement: FIGURE S2 — Alterations in lipid mediators of inflammation resolution (LMIR) in response to AgNP exposure. Lipids are tentative attributions based on flow injection and multiple reaction monitoring. Alterations in (A) linolenic acid, (B) stearidonic acid, and (C) eicosatetraenoic acid were observed due to MetS in response to AgNP exposure. ∗ denotes lipids significantly reduced only in MetS in response to AgNP exposure, while $ denotes lipids significantly reduced in exposed MetS compared to exposed healthy mice, and # denotes significant differences due to statin treatment (p < 0.05). [file Image_2.tiff]

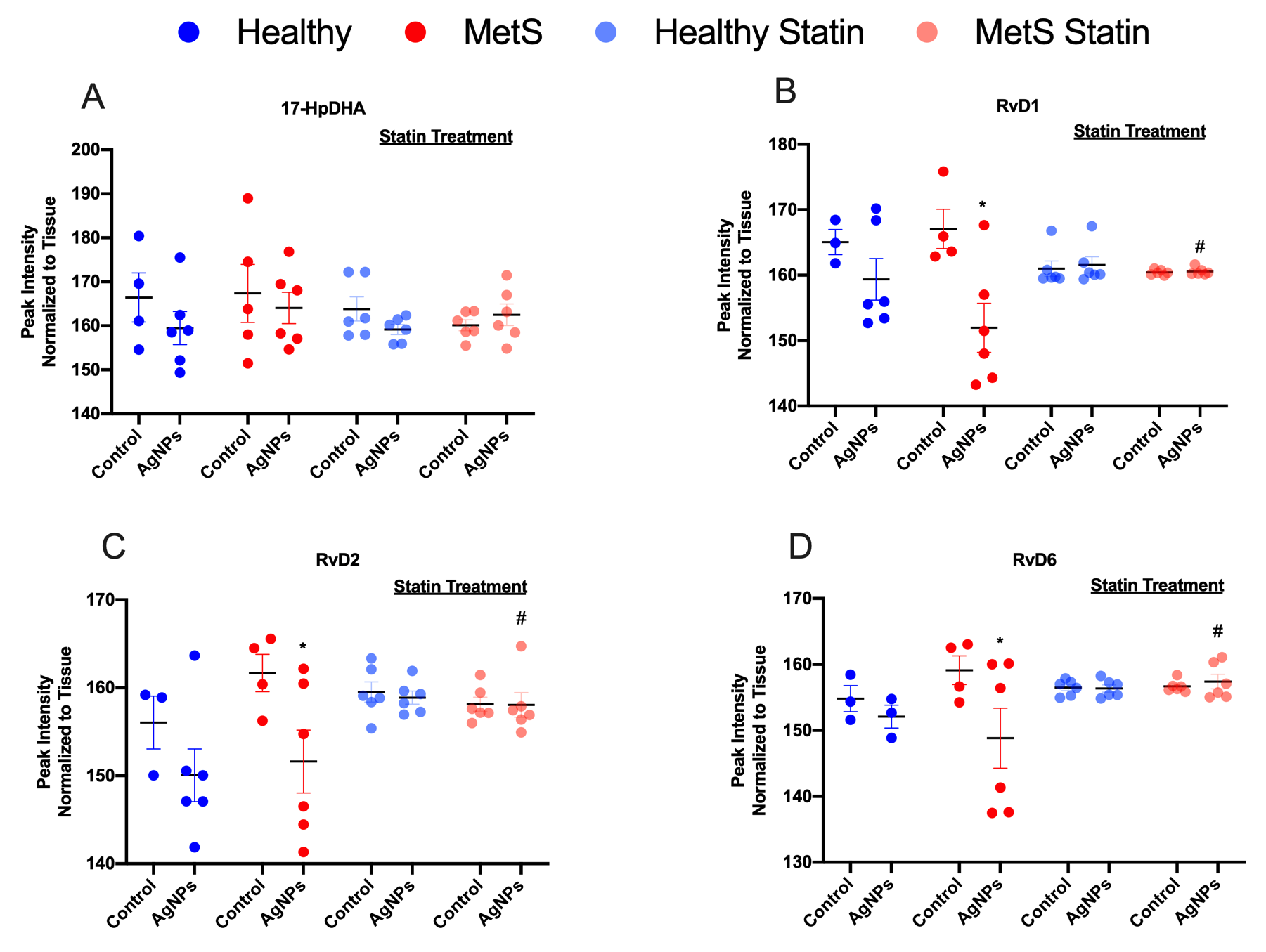

Supplement: FIGURE S3 — Assessment of docosahexaenoic acid (DHA)-derived lipid mediators of inflammatory resolution (LMIR). Lipids are tentative attributions based on flow injection and multiple reaction monitoring. Alterations in (A) 17-HpDHA, (B) RvD1, (C) RvD2, and (D) RvD6 were observed due to MetS in response to AgNP exposure. ∗ denotes lipids significantly reduced in MetS in response to AgNP exposure, and # denotes significant differences due to statin treatment (p < 0.05). [file Image_3.tiff]
